# Beyond degree and betweenness centrality: Alternative topological measures to predict viral targets

**DOI:** 10.1371/journal.pone.0197595

**Published:** 2018-05-24

**Authors:** Prajwal Devkota, Matt C. Danzi, Stefan Wuchty

**Affiliations:** 1 Dept. of Computer Science, Univ. of Miami, Coral Gables, FL, United States of America; 2 The Miami Project to Cure Paralysis, Miller School of Medicine, University of Miami, Miami, FL, United States of America; 3 Center for Computational Science, Univ. of Miami, Coral Gables, FL, United States of America; 4 Dept. of Biology, Univ. of Miami, Coral Gables, FL, United States of America; 5 Sylvester Comprehensive Cancer Center, Miller School of Medicine, University of Miami, Miami, FL, United States of America; Universitatsmedizin Greifswald, GERMANY

## Abstract

The availability of large-scale screens of host-virus interaction interfaces enabled the topological analysis of viral protein targets of the host. In particular, host proteins that bind viral proteins are generally hubs and proteins with high betweenness centrality. Recently, other topological measures were introduced that a virus may tap to infect a host cell. Utilizing experimentally determined sets of human protein targets from Herpes, Hepatitis, HIV and Influenza, we pooled molecular interactions between proteins from different pathway databases. Apart from a protein’s degree and betweenness centrality, we considered a protein’s pathway participation, ability to topologically control a network and protein PageRank index. In particular, we found that proteins with increasing values of such measures tend to accumulate viral targets and distinguish viral targets from non-targets. Furthermore, all such topological measures strongly correlate with the occurrence of a given protein in different pathways. Building a random forest classifier that is based on such topological measures, we found that protein PageRank index had the highest impact on the classification of viral (non-)targets while proteins' ability to topologically control an interaction network played the least important role.

## Introduction

The arrival of high-throughput screens that allow the generation of large datasets of protein interactions has enabled scientists to comprehensively understand the ways different proteins interact with each other within and between cells. Over the last decade, protein interaction interfaces of several human pathogens and their human host cells have been experimentally determined [1–7], indicating physical interactions between viral and human host proteins. In addition, interaction interfaces of several other pathogens such as bacteriophages [8] and parasites [9] have been investigated as well. Various RNAi screens have additionally revealed sets of human proteins required by different human viruses to infect their host cells [10–12]. While they usually do not directly interact with viral proteins, required genes play a decisive role in the viral infection process as removal or loss of function prevents the virus from accomplishing its task. Such sets have also been determined in bacteriophage-host systems [13, 14].

The understanding of their topological influence in a host-specific molecular interaction network helps us to gain insight on why pathogens choose certain host proteins as targets. Therefore, the availability of such large interaction sets between human host and viral proteins has already prompted researchers to investigate the characteristics of these pathogen-host interfaces as well as sets of virus-specific genes that are required for the corresponding infection process [15–24]. Generally, viral proteins tend to target hubs and bottleneck proteins in the underlying host protein interaction network. Notably, such parameters were applied to predict potential viral targets using machine-learning approaches [25–28].

Apart from such topological measures in protein interaction networks, pathway information has also been tapped to predict interactions between viral and host proteins [29, 30]. Notably, topological features can be used for pathway based impact factor analysis [31], using a variation of Google’s PageRank algorithm. Such observations suggest that topological features may serve as reliable predictors of potential viral protein targets. Here, we used different topological measures beyond simple degree and betweenness centrality to investigate their propensity to characterize viral targets. We utilized a molecular protein interaction network that has been assembled from different molecular pathway databases. Apart from degree and betweenness centrality we considered the number of times a protein occurs in a pathway, the protein PageRank index and the ability of proteins to topologically control the underlying network. We found that all measures correlated with increasing propensity of proteins to be targeted by different human viruses. Furthermore, we showed that such measures have different impact on the ability to predict viral targets as well as were independent from the underlying pathway and viral target information.

## Results and discussion

**Fig 1:**
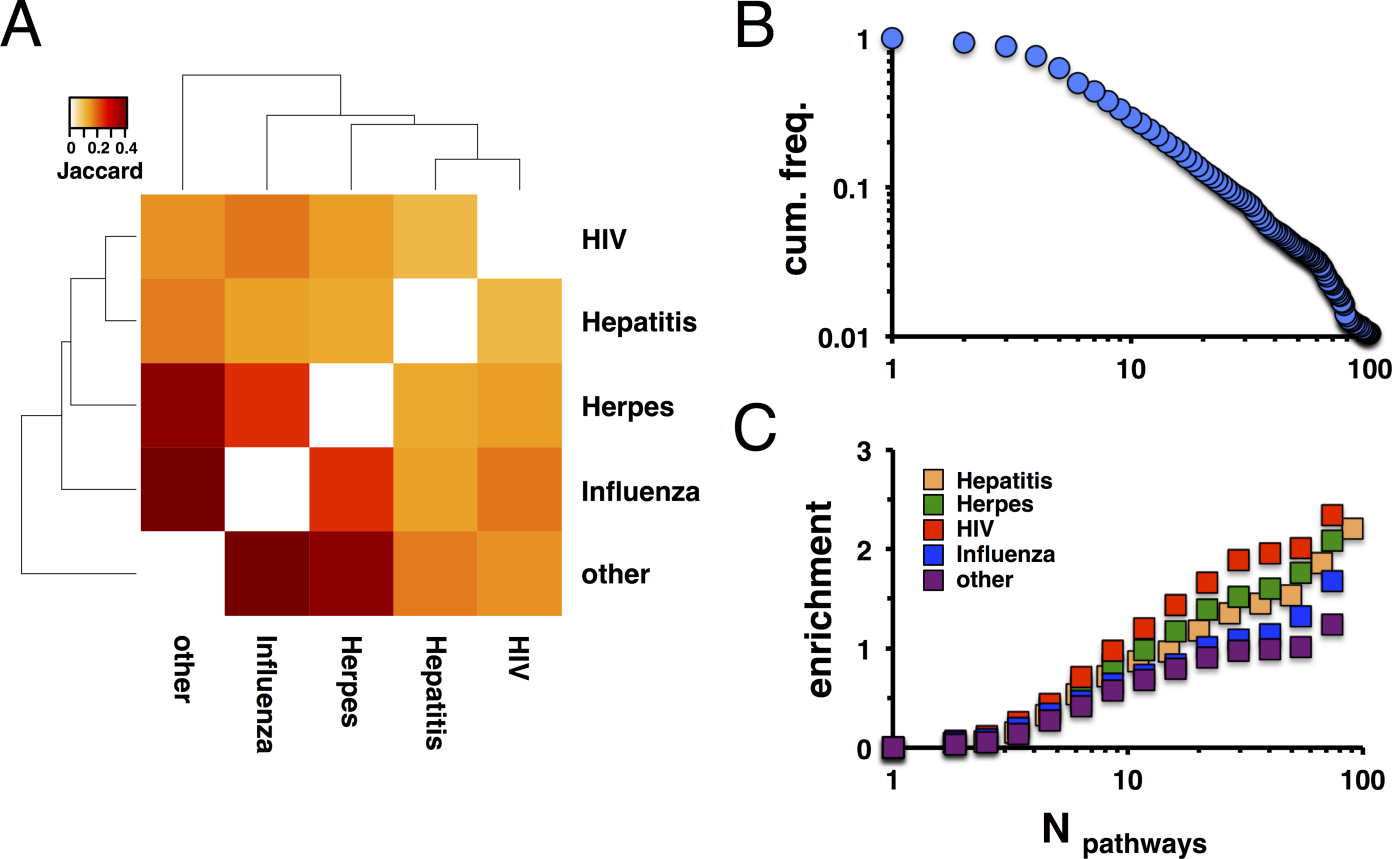
Enrichment of human targets of Hepatitis, Herpes, HIV, Influenza and other viruses as a function of pathway participation. In **(A)**, we determined the overlap between sets of viral targets of Hepatitis, Herpes, HIV, Influenza and other viruses, determining the corresponding Jaccard indices. We observed that targets of Herpes, Influenza and other viruses overlapped strongly. In **(B)**, we determined the occurrence of proteins in different pathways. The cumulative frequency distribution thus obtained featured a heavy tail, indicating that a small minority of proteins appeared in a large number of pathways and *vice versa*. In **(C)**, we determined the enrichment of viral targets as a function of targeted protein’s occurrence in different pathways by randomly sampling viral target sets 10,000 times. We found that targets appeared in an increasing number of pathways.

We collected sets of human proteins that were targeted by Hepatitis, Herpes simplex, Influenza, HIV, and remaining viruses as of the HPIDB database [32]. Importantly, these four human-infecting viruses are very different in their taxonomy, nucleotide content, and mode of infection. Counting the number of shared proteins, we determined the corresponding Jaccard index of overlapping target gene sets (**Fig 1A**). We generally observed that virus-specific target sets moderately overlapped, indicating highest overlap between Influenza, Herpes and targets of remaining viruses.

To determine topological parameters of a large network of molecular interactions, we collected human pathways from the KEGG [33], Reactome [34], Biocarta and NCI PID [35] databases. In particular, we used the graphite tool [36] to parse pathway information, allowing us to represent each pathway as a network of directed interactions. Pooling all interactions in each pathway from all pathway sources we obtained a network of 10,981 human proteins that were involved in 622,056 directed interactions. As for descriptive statistics, **Fig 1B** indicated that the distribution of occurrences of proteins in different pathways has a fat tail. Such an observation suggested that a minority of proteins participated in an increasing number of pathways and *vice versa*. Investigating networks that were generated from considered databases separately, we obtained similar results (**S1 Fig**). Assuming that a minority of proteins allows a broad reach into a variety of pathways, we hypothesized that viruses may potentially tap such a characteristic. Randomizing sets of viral targets 10,000 times, we determined the enrichment of viral targets in sets of proteins that appear in an increasing number of pathways. **Fig 1C** suggested that all viruses preferentially targeted proteins that reached into an increasing number of pathways. Notably, such an observation was independent from the underlying pathway data as we observed similar results, when we separately considered KEGG, Reactome and Biocarta/NCI data (**S2 Fig**).

**Fig 2:**
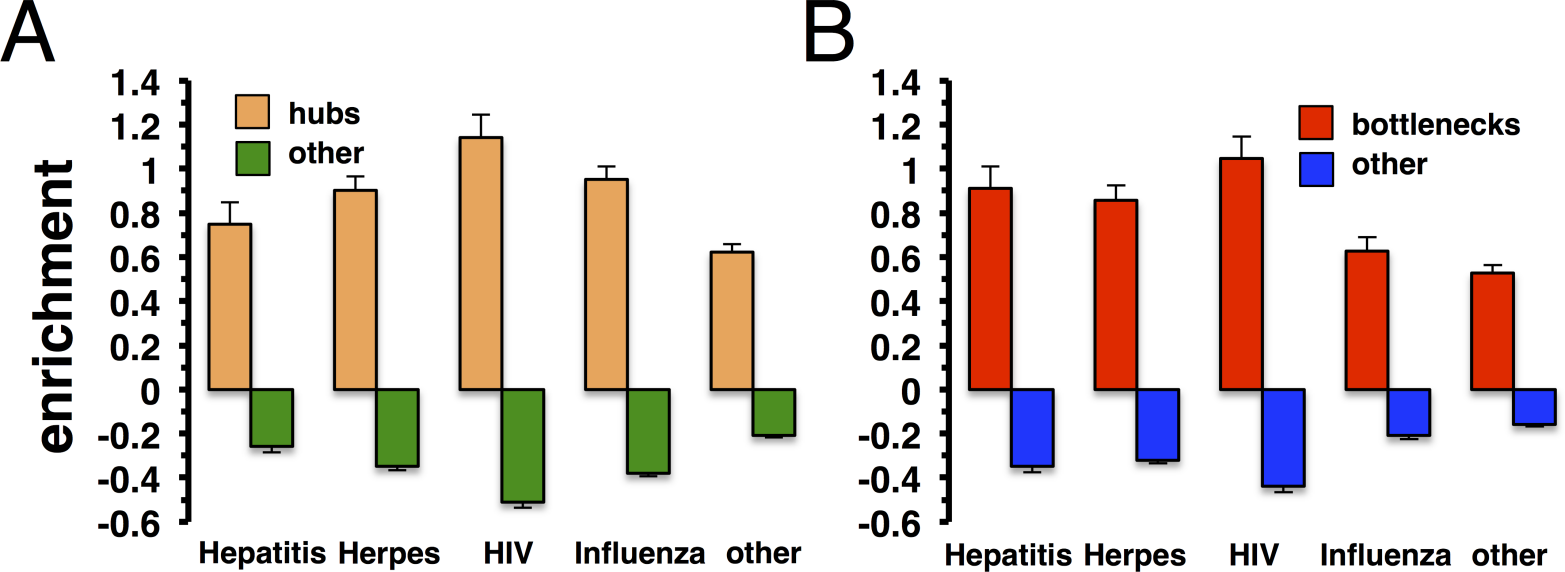
Enrichment of viral targets in sets of hubs and bottleneck nodes. **(A)** Defining the top 20% of most connected proteins as hubs, we determined the enrichment of targets of Hepatitis, Herpes, HIV, Influenza and other viruses in such sets. Randomly sampling sets of targeted proteins 10,000 times, we observed that targets were significantly enriched in the set of hubs and *vice versa* (P<10^−4^). In **(B)**, we defined the top 20% of proteins with highest betweeness as bottleneck nodes. Randomly sampling sets of targeted proteins 10,000 times, we found that bottleneck protein preferably were targeted by viruses while the opposite held for non-bottleneck proteins (P<10^−4^).

In a similar vein, we determined the enrichment of viral targets in sets of highly connected and highly central proteins. Considering each interaction undirected we defined a set of hub genes as the top 20% of highest connected proteins. In turn, we calculated betweenness centrality of each protein in the underlying directed network of protein interactions and defined the top 20% of proteins with highest centrality as a set of bottleneck proteins. In **Fig 2A**, we randomly sampled targets of Hepatitis, Herpes, HIV, Influenza and other viruses 10,000 times. Determining their enrichment, we clearly observed that hub proteins were significantly enriched with viral targets and *vice versa* (P<10^−4^). Similarly, **Fig 2B** indicated that bottlenecks were significantly targeted by viruses (P<10^−4^) while the opposite held for non-bottleneck proteins. To determine the dependence of our results from different pathway sources, we found that our observations held independently from different pathway data (**S3 Fig)**.

**Fig 3:**
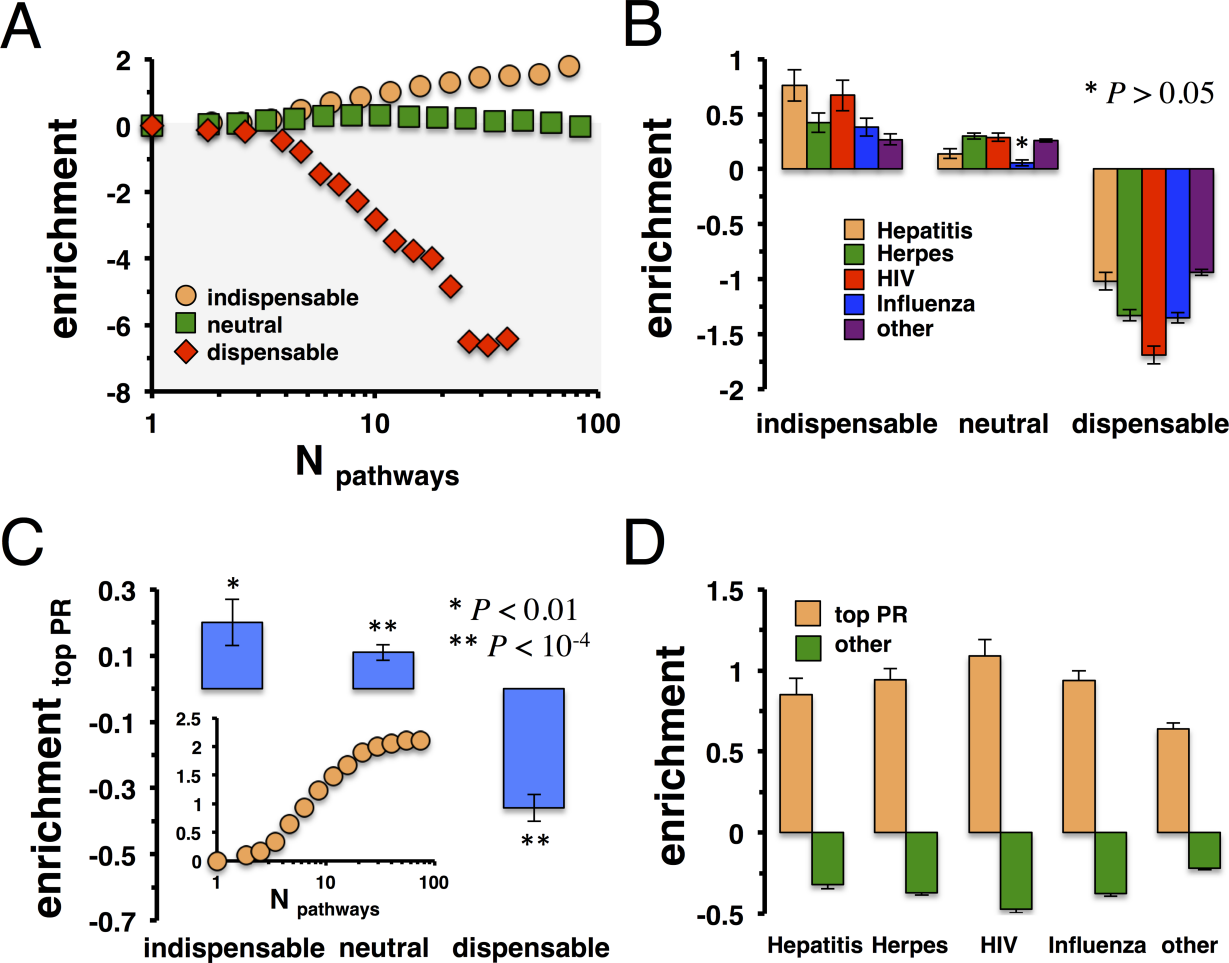
Network controllers and proteins with high protein page rank are enriched with viral targets. In **(A)** we determined indispensable, neutral and dispensable proteins in the underlying protein interaction network. Randomizing such sets 10,000 times, we observed that proteins that are indispensable for the control of the underlying network preferably occurred in an increasing number of pathways. In turn, we found the opposite for dispensable proteins. **(B)** Randomizing sets of proteins that are targeted by Hepatitis, Herpes, HIV, Influenza and other viruses 10,000 times, we observed that indispensable proteins are preferably targeted by viruses (P<10^−4^) while the opposite held for dispensable nodes. **(C)** Randomizing the set of top PageRank proteins, we determined their enrichment in sets of indispensable, neutral and dispensable proteins. We observed that indispensable and neutral nodes significantly accumulated top PageRank proteins. In the inset, we observed that proteins in an increasing number of pathways were enriched with top PageRank proteins. **(D)** Randomizing sets of targets of Hepatitis, Herpes, HIV, Influenza and other viruses 10,000 times we observed that proteins with highest protein PageRank were significantly targeted (P<10^−4^).

Another topological characteristic that may be tapped by viruses were nodes that topologically controlled the underlying network. In particular, we calculated maximum-matching configurations in a bipartite representation of directed links in the underlying network of molecular interactions. We defined a node as indispensable for the topological control of the underlying network if the cardinality of the set of controllers (i.e. indispensable proteins) increased when we deleted the considered node. If the number of controllers was unchanged we considered the deleted node neutral. Furthermore, a node was defined as dispensable if less controlling nodes were found upon deletion [37]. In particular, we found 1,393 indispensable, 6,350 neutral and 3,238 dispensable proteins in the underlying network of directed interactions. We randomly sampled sets of (in-)dispensable and neutral proteins 10,000 times and determined their mean enrichment in bins of proteins that occurred in a certain number of pathways. **Fig 3A** suggested that indispensable proteins preferably appeared in an increasing number of pathways. While neutral proteins did not show a significant trend, dispensable proteins appeared diluted among proteins that appeared in an increasing number of pathways. Furthermore, such observations were independent from the underlying pathway data (**S4 Fig**). As a corollary, we determined the enrichment of viral targets in sets of (in-)dispensable and neutral proteins. In **Fig 3B**, we randomly sampled targets of Hepatitis, Herpes, HIV, Influenza and other viruses 10,000 times and found that indispensable nodes were significantly enriched with viral targets (P<10^−4^). In turn, the opposite held for dispensable proteins, results that were independent from the underlying pathway data (**S5 Fig**).

As another global topological parameter we considered the protein PageRank index, defined as the probability that a random walker ended up at a given protein. Notably, such a measure is based on the original Google PageRank, which reflects the probability that a random walker reaches a page following hyperlinks on web pages. We hypothesized that proteins that unified information flow from different parts of the network may be preferable targets of viruses. Determining the protein PageRank of each protein in the underlying directed network of molecular interactions, we considered the top 20% of proteins with highest protein PageRank. Randomly sampling such sets of proteins 10,000 times, we determined their enrichment in sets of indispensable, neutral and dispensable proteins. **Fig 3C** suggests that indispensable and neutral proteins were significantly enriched with top PageRank proteins, while we found the opposite, considering dispensable proteins (P < 10^−4^). Furthermore, we determined the enrichment of top PageRank proteins as a function of their corresponding appearance in pathways. In the inset of **Fig 3C**, we clearly observed that such proteins predominately appeared in sets of proteins that occurred in an increasing number of pathways. As a corollary, we investigated the ability of top PageRank proteins to accumulate viral targets. Randomizing such sets of virus-specific targets 10,000 times, we found that viruses preferably targeted proteins with a high PageRank index and *vice versa* (**Fig 3D**, P<10^−4^). Notably, such observations were independent from the utilized pathway data (**S6 Fig**).

**Fig 4:**
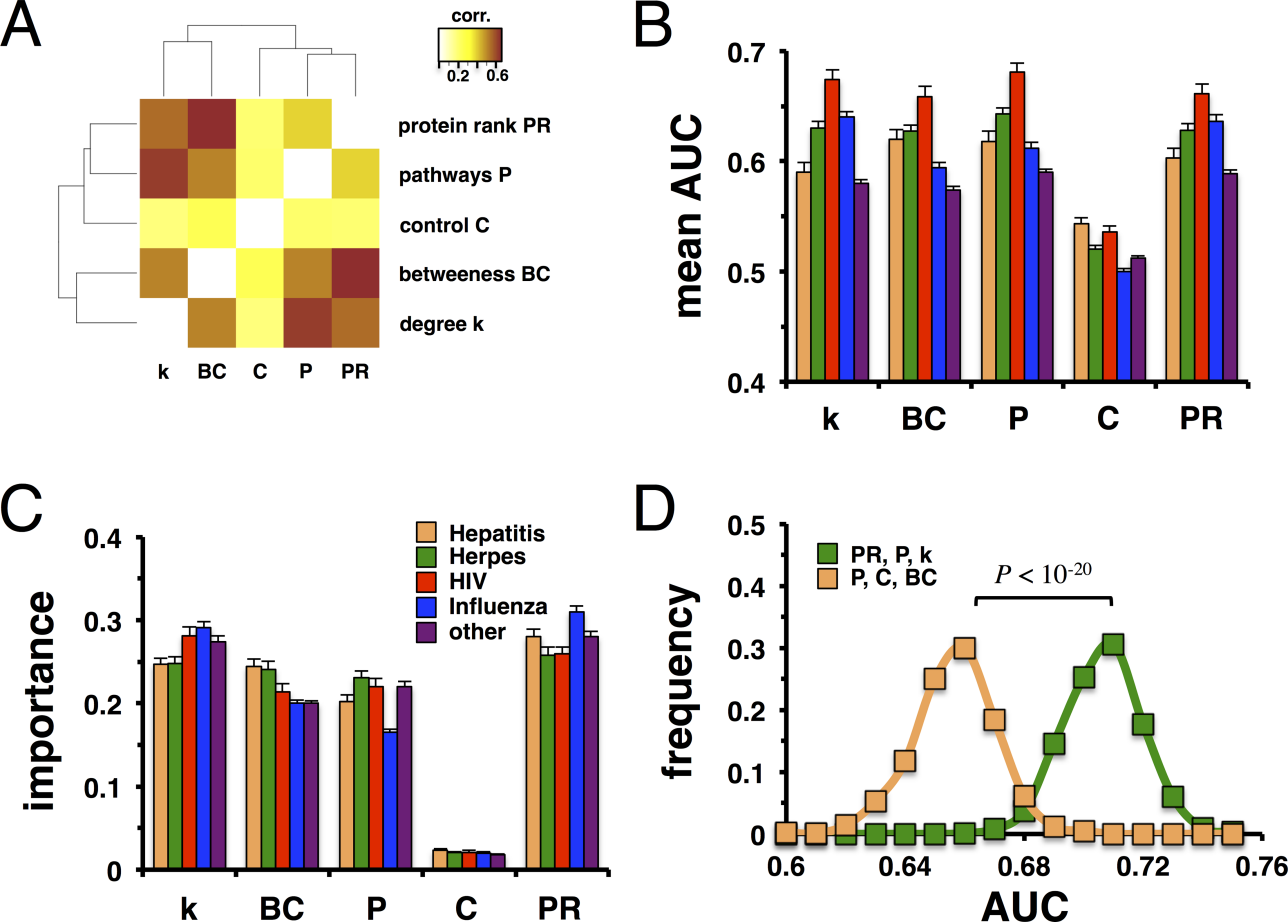
Prediction of viral targets. **(A)** The heatmap indicated Pearson correlation values between the distributions of degree, betweenness centrality, number of pathways a protein is involved in, protein PageRank index and indispensability of a protein. Notably, degree, protein PageRank, betweenness centrality and appearance in pathways appeared best correlated while indispensability of proteins showed lowest levels of correlation with other topological measures. **(B)** Considering target sets of Hepatitis, Herpes, HIV, Influenza and other viruses, we randomly sampled sets of non-targeted proteins of equal size. Determining the area under the ROC curves (AUC), we observed that protein PageRank index and pathway participation of a protein allowed the most thorough classification of (non-)targets. **(C)** As a corollary, we utilized all five topological measures to predict viral targets using a random forest. We found that protein PageRank had the highest impact on the classification process, a result that was independent of the underlying virus. In **(D)**, we randomly sampled sets of non-targeted proteins 1,000 times that were equal in size to the set of HIV targets and determined the area under the ROC curve (AUC) of the classification process with a random forest. In particular, we predicted if a protein was (not) targeted as a function of the three most (protein PageRank index, degree and pathway appearance) and least important topological features (betweenness centrality, pathway appearance, control). Notably, the distributions of AUC values thus obtained were statistically significant (Student’s t-test, P < 10^−20^), suggesting that most important features allowed a significantly better classification result.

Given our frequent observation that topological parameters correlated with the underlying proteins' occurrence in an increasing number of pathways, we determined a matrix composed of Pearson correlation coefficients between all pairs of topological measures. The heatmap in **Fig 4A** generally suggests that all topological measures are reasonably correlated while degree, betweenness centrality, protein PageRank index and pathway appearance showed strongest correlations between each other. As for control features of proteins, we only accounted for proteins that were labeled indispensable. In turn, indispensability of proteins appeared the least correlated with other topological measures. To corroborate our results, we determined the corresponding heatmaps of correlations between network parameters in networks that have been obtained from different pathway data, separately (**S7 Fig)**. We observed that degree and appearance in pathways generally showed highest degree of correlation to other network parameters in the networks while indispensability of proteins showed lowest levels of correlation.

Considering all topological measurements separately, we utilized target sets of Hepatitis, Herpes, HIV, Influenza and other viruses as positive training data, and randomly sampled sets of non-targeted proteins as negative training data sets of equal size. In **Fig 4B**, we determined the mean area under ROC curves (AUC) for each pair of topological measure and virus, using 1,000 random samples of negative training sets. Independently from the type of virus, we found that protein rank and a protein’s appearance in pathways allowed the most thorough classification of targets. Notably, such an observation is corroborated when we considered different pathway data separately (**S8 Fig**). To determine the topological measure of the underlying interaction network that has the highest impact on the classification process, we applied a random forest classifier. Utilizing all five topological measures to distinguish between randomly sampled sets of non-targets as negative training and targeted proteins as positive training data we calculated the mean importance of each characteristic. In particular, the importance of a parameter is determined by the change in classification accuracy if a given parameter is omitted. On average, protein rank had highest importance followed by degree and betweenness centrality (**Fig 4C**). Notably, such an observation was independent of the virus target sets as well as the underlying pathway data (**S9 Fig**). To investigate the impact of different topological characteristics further we determined area under the ROC curves using different combinations. In **Fig 4D**, we considered protein targets of HIV and randomly sampled non-targeted protein sets of equal size. Using a random forest algorithm, we predicted if a protein was (not) targeted as a function of the three most and least important topological features. As the random forest algorithm sampled a subset of the underlying training data to establish decision trees, remaining training data were used as testing data. Predicting targets through out-of-bag samples, we obtained best classification results with most important features (protein PageRank index, appearance in pathways and a protein’s degree). Notably, classification differences compared to the three least important measures (betweenness centrality, protein indispensability and pathway appearance) were statistically significant (Student’s t-test, P<10^−20^), a result that largely held for the prediction of targets of Hepatitis, Herpes, influenza and other viruses as well (**S10 Fig**).

## Conclusions

Previous approaches tout the role of the simple degree and betweenness centrality as topological features in molecular interaction networks that viruses and other pathogens tap. Here, we investigated other more recent topological parameters that previously have not been considered as pathogen relevant. Specifically, we based our considerations on a directed molecular interaction network that was compiled from all pathways of the KEGG, Reactome and Biocarta/NCI databases. To lay the groundwork, we confirmed that targeted proteins were preferably hubs and bottleneck proteins. Furthermore, we considered the number of pathways that a protein appeared in as a potential pathogen feature, as viruses may thoroughly penetrate a host system by reaching into many different pathways. Indeed, we observed that viral targets were enriched with proteins that appeared in an increasing number of pathways. Such an observation appeared plausible, assuming that viruses need to utilize their limited protein repertoire to secure maximum impact on the host cell through reaching into a large number of pathways, an objective that is further aided by hubs and bottleneck proteins.

Another global topological parameter that we considered was the protein PageRank index, defined as the probability that a random walker ended up at a given protein. Notably, such a measure was based on the original Google PageRank, which reflected the probability that a random walker reaches a page following hyperlinks on web pages. As high PageRank indicated proteins occurred in an increasing number of pathways, we hypothesized that proteins that unify information flow from different parts of the network may be viral targets. Indeed, we observed that proteins with a high protein PageRank were indeed enriched with proteins that viruses bind.

Recently, different measures for a node’s ability to control a network were introduced. Here, we determined the role of proteins in terms of their contribution to topologically control a network by distinguishing indispensable, neutral and dispensable proteins in the underlying network. Notably, a protein's role in the topological control of the underlying network also correlated with the occurrence of such proteins in an increasing number of pathways, suggesting that viruses may tap such a characteristic. Indeed, we found that indispensable proteins were prime viral targets while dispensable proteins were hardly found targeted.

The underlying molecular interaction network was based on the pooled interactions of different pathway databases. Notably, all topological characteristics of proteins were well correlated with their corresponding participation in different pathways. Such an observation suggested that centrality in terms of degree, betweenness, protein rank and ability to control a network was a function of increased pathway occurrence. We also investigated how the different measures stacked up in their ability to predict potential viral targets. Instead of a protein’s appearance in different pathways, we surprisingly found that a protein PageRank predicted viral targets best when we considered each measure in isolation. Such a result is corroborated when we considered the impact of each feature, training a random forest algorithm with sets of virus specific targets. In turn, network control had least impact on the classification process. Putting the most important features together we obtained significantly better prediction results compared to a random forest classifier that was built on the least important topological measures.

Notably, we observed that our results were independent from the viruses considered, suggesting that viruses generally tap similar topological features to control a human host cell. Furthermore, our observations were independent from the underlying sources of pathway information, suggesting that the way the underlying molecular interactions were obtained do not preordain the outcome of our analysis and prediction even though the underlying network was based on the pool of all pathways.

While our results were encouraging in terms of finding topological features that are potentially tapped by viral pathogens, such characteristics may also be indicative of other non-targeted proteins with certain functions, limiting the assumption that viral targets can be determined on topological grounds alone. Viral targets tend to closely interact with proteins that are important for the infection of the underlying viral pathogen as well as essential proteins [24]. In particular, recent analyses revealed that host genes that were critical for the infection of the HIV virus may be direct targets as well [38], suggesting that their topological characteristics were similar [17]. Furthermore, a virus’ propensity to interact with hub and bottleneck proteins appeared to be a consequence of interactions with particular cellular functions as well, rather than being a direct effect of network topological properties alone [39].

## Materials and methods

### Pathway information

We utilized 294 molecular human pathways from the KEGG database [33], 1,924 pathways from Reactome [34] and 459 pathways from Biocarta and the NCI PID database [35]. Interactions between proteins were parsed with graphite [36], allowing us to represent each pathway as a directed, unweighted network. Pooling such directed interactions of all pathways we created a network of 10,981 proteins embedded in 622,056 directed interactions. As for database specific networks, we obtained a web of 9,878 proteins and 510,626 directed interactions from Reactome pathways. While we found 5,424 proteins that were linked by 114,123 interactions using KEGG pathway data, we obtained 2,737 proteins and 36,204 interactions from Biocarta/PID databases.

### Virus-host interactions

Collecting data from the HPIDB database, we obtained 988 human proteins that were targeted by the Hepatitis C virus, as well as 2,157 targets of the Herpes simplex virus, 872 targets of HIV-1 and 2,358 targets of the Influenza A virus [32]. In addition, we accounted for 5,619 targets of other, remaining human viruses.

### Enrichment analysis

Binning proteins with a certain characteristic *d* (e.g. viral target) we calculated the fraction of proteins that had a feature *i* (e.g. bottleneck protein) in each group *d*, *f*_*i*_*(d)*. As a null model we randomly sampled protein sets with feature *i* of the same size 10,000 times and calculated the corresponding random fraction, *f*_*i*,*r*_ *(d)*. The enrichment/depletion of proteins with feature *i* in a group *d* was then defined as *E_i_*(*d*) = *lg*_2_(*f_i_*(*d*)/*f_i,r_*(*d*)). As a variation of this approach, we calculated the enrichment of proteins with a characteristic *d* as a function of a topological parameter *t* (e.g. number of pathways a protein occurs in). Specifically, we binned proteins in groups *N*_≥*t*_ where each protein was characterized by a parameter ≥ *t* and calculated the corresponding number of proteins with characteristic *d*, *N*_*d*,≥*t*_. Randomly sampling genes we defined $E_{d,\geq t}={lg}_{2}(N_{d,\geq t}/N_{d,\geq t}^{r})$ as the enrichment of proteins with *d* where $N_{d,\geq t}^{r}$ was the corresponding random number of proteins with characteristic *d* among all *N*_≥*t*_ proteins in the corresponding bin. After averaging *E*_*i*_ over 10,000 randomizations *E*_*d*_ *>0* pointed to an enrichment and *vice versa*, while *E*_*i*_ *~ 0* indicated a random process [40].

### Protein’s PageRank index

In our directed network of protein interactions, we calculated each protein’s PageRank index, representing a stationary limit probability that a random walker will reach the corresponding protein [41, 42]. We defined the protein PageRank index as *PR*(*g*) = (1 − *d*)*N*^−1^ + ∑_*u*∈*U*(*g*)_*PR*(*u*)/*N_ds_*(*u*), where *U(g)* is the set of upstream proteins of protein *g*, *N*_*ds*_*(u)* is the number of proteins downstream of protein *u* and *N* is the total number of proteins in the underlying network. Furthermore, *d* is a damping factor that has been set to 0.85. Based on this measure, we defined a set of protein with top protein PageRank as 20% highest ranking proteins.

### Betweenness centrality

As a global measure of its centrality, we calculated node betweenness, indicating a node’s appearance in shortest paths through the whole network. In particular, we defined betweenness centrality *c*_*B*_ of a node *v* as *c_B_* = ∑_*s*≠*t*≠*v*∈*V*_*σ_st_*(*v*)/*σ_st_*, where *σ_st_* was the number of shortest paths between proteins *s* and *t*. Furthermore, *σ_st_*(*v*) was the number of shortest paths running through *v*. Based on this measure, we defined a set of bottleneck proteins as the top 20% of proteins with highest betweenness.

### Random forests

Random Forests (RF) is an ensemble learning method [43] where classification trees are constructed with *N* different bootstrap samples of the data (‘bagging’). In addition, random forests change how classification trees are constructed by splitting each node, using the best among a subset of *M* predictors randomly chosen at that node (‘boosting’), and new data is predicted by aggregating the predictions of *N* trees. The Variable Importance is calculated using Breiman-Cutler permutation [43], recording the prediction error on out-of-bag (OOB) data in each tree of a random forest. OOB cases are then randomly permuted for a variable *x*, and the prediction error is recorded. Variable importance is then determined based on the difference between the perturbed and unperturbed error rate and averaged over all trees. We used a Python implementation of the random forest algorithm as of the sklearn package.

### Network controllers

Utilizing the directed network of protein interactions, we determined nodes that are relevant for the topological control of networks by applying a maximum matching algorithm [44] that aims at matching each directed interaction. In particular, directed interactions are represented as a bipartite graph, where proteins that cannot be matched are considered network controllers. However, many equivalent solutions for the maximum matching problem exist. Therefore, we defined sets of proteins as indispensable if the number of unmatched nodes increased when we deleted the underlying node from the network. In turn, we found a dispensable node if the number of unmatched nodes declined upon deletion of the considered node. Neutral nodes did not change the number of unmatched nodes when they were deleted [37].

## Supporting information

S1 Fig Frequency distributions of proteins pathway participation. Utilizing networks that were obtained from pathways in Reactome, Kegg, and Biocarta/NCI, we observed heavy tails in the frequency distributions of the numbers of pathways that proteins occur in.
(TIFF)

S2 Fig Enrichment of viral targets as a function of pathway participation. We determined the enrichment of viral targets as a function of targeted protein’s occurrence in different pathways. Randomly sampling viral target sets 10,000 times, we found that targets appeared to be involved in an increasing number of pathways, a result that held for KEGG, Reactome and Biocarta/NCI pathways, respectively.
(TIFF)

S3 Fig Enrichment of viral targets in sets of hubs and bottlenecks. Defining the top 20% of most connected proteins as hubs and top 20% of proteins with highest betweenness centrality as bottleneck proteins, we determined the enrichment of viral targets in such sets. Randomly sampling viral target sets 10,000 times, we generally observed that viral targets appeared enriched in sets of hubs and bottleneck nodes when we considered networks from different pathway sources, separately.
(TIFF)

S4 Fig Enrichment of (in-)dispensable and neutral proteins. We randomly sampled sets of (in-)dispensable and neutral proteins and determined their enrichment in bins of proteins that occur in a certain number of KEGG, Reactome and Biocarta/NCI pathways. In all cases, we observed that indispensable proteins preferably appeared in an increasing number of pathways. Neutral proteins did not show any significant trend while dispensable proteins appeared diluted among proteins that appeared in an increasing number of pathways.
(TIFF)

S5 Fig Network controllers are enriched with viral targets. Randomizing sets of proteins that are targeted by Hepatitis, Herpes, HIV, Influenza and other viruses 10,000 times, we observed that indispensable proteins were preferably targeted by viruses (P<10^−4^) while the opposite held for dispensable nodes. Such observations held irrespective of the underlying pathway data source.
(TIFF)

S6 Fig Proteins with high protein PageRank index are enriched with viral targets. Randomizing sets of viral targets 10,000 times we determined their enrichment in sets of the top 20% of proteins with highest PageRank index. We observed that proteins with top PageRank were significantly enriched with viral targets and *vice versa* (P < 0.01). Notably, such observations were independent from pathway specific data.
(TIFF)

S7 Fig Correlation between topological measures. The heatmap indicated Pearson correlation values between the distributions of degree, betweenness centrality, number of pathways a protein is involved in, protein PageRank and indispensability of a protein using different pathway information.
(TIFF)

S8 Fig Classifier performance of different topological measures. Considering target sets of Hepatitis, Herpes, HIV, Influenza and other viruses, we randomly sampled sets of non-targeted proteins of equal size. In comparison to betweenness centrality (BC) and protein’s indispensability (C) we observed that protein PageRank index (PR), pathway participation (P) of a protein and a proteins degree (k) allowed the most thorough classification of (non-)targets. Such observations were independent from the pathway information used.
(TIFF)

S9 Fig Importance of topological measures. We utilized all five topological measures to predict viral targets using a random forest. We found that protein PageRank had the highest impact on the classification process, a result that was independent of the underlying virus and pathway information.
(TIFF)

S10 Fig Performance of random forest classifier, predicting viral targets. Focusing on proteins that are targeted by Hepatitis, Herpes, Influenza and other viruses, we randomly sampled non-targeted proteins of equal size 1,000 times. Furthermore, we used a random forest trained with the three most and least important features. In all cases, we found that the AUC curves obtained from the most important features allowed a significantly better classification (Student’s t-test, P < 10^−20^).
(TIFF)
