# Supplementary figures and images for: Beyond degree and betweenness centrality: Alternative topological measures to predict viral targets
Source: PLoS One. 2018 May 24;13(5):e0197595. doi: 10.1371/journal.pone.0197595 (PMC5967884; doi:10.1371/journal.pone.0197595)

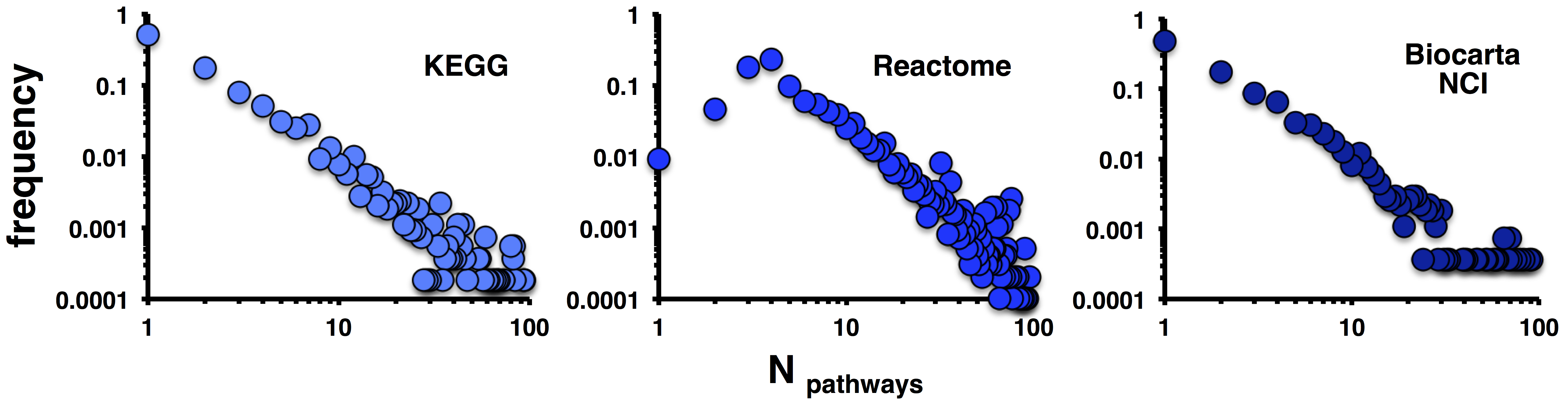

Supplement: S1 Fig — Utilizing networks that were obtained from pathways in Reactome, Kegg, and Biocarta/NCI, we observed heavy tails in the frequency distributions of the numbers of pathways that proteins occur in. (TIFF) [file pone.0197595.s001.tiff]

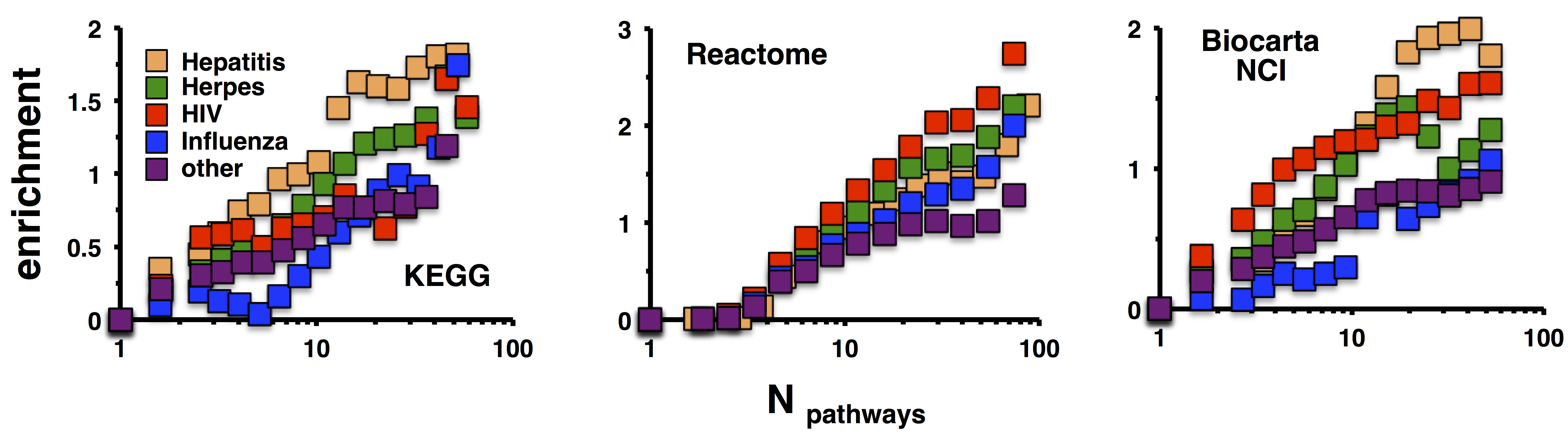

Supplement: S2 Fig — We determined the enrichment of viral targets as a function of targeted protein’s occurrence in different pathways. Randomly sampling viral target sets 10,000 times, we found that targets appeared to be involved in an increasing number of pathways, a result that held for KEGG, Reactome and Biocarta/NCI pathways, respectively. (TIFF) [file pone.0197595.s002.tiff]

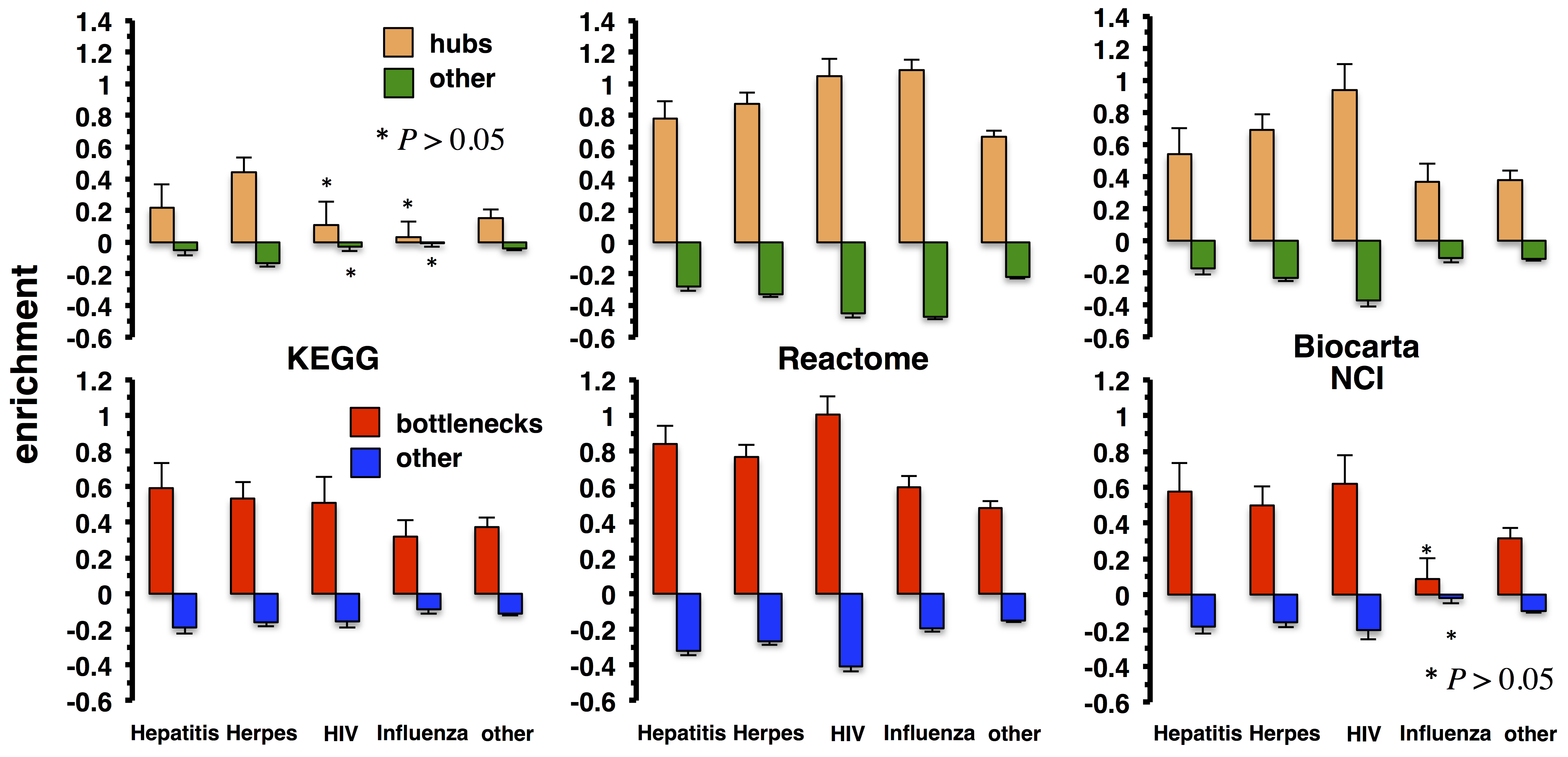

Supplement: S3 Fig — Defining the top 20% of most connected proteins as hubs and top 20% of proteins with highest betweenness centrality as bottleneck proteins, we determined the enrichment of viral targets in such sets. Randomly sampling viral target sets 10,000 times, we generally observed that viral targets appeared enriched in sets of hubs and bottleneck nodes when we considered networks from different pathway sources, separately. (TIFF) [file pone.0197595.s003.tiff]

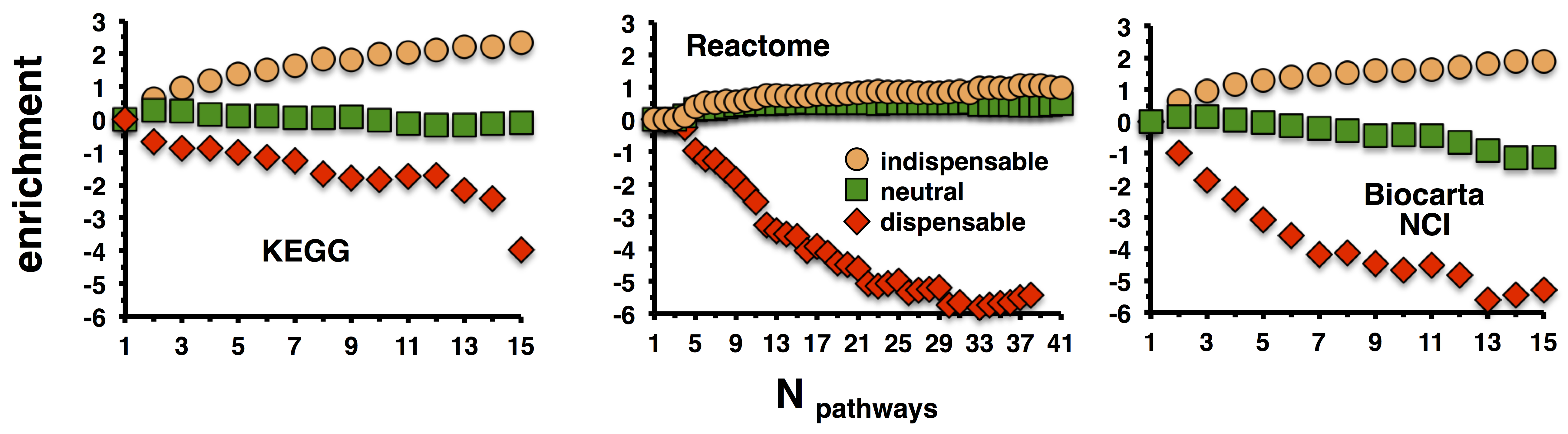

Supplement: S4 Fig — We randomly sampled sets of (in-)dispensable and neutral proteins and determined their enrichment in bins of proteins that occur in a certain number of KEGG, Reactome and Biocarta/NCI pathways. In all cases, we observed that indispensable proteins preferably appeared in an increasing number of pathways. Neutral proteins did not show any significant trend while dispensable proteins appeared diluted among proteins that appeared in an increasing number of pathways. (TIFF) [file pone.0197595.s004.tiff]

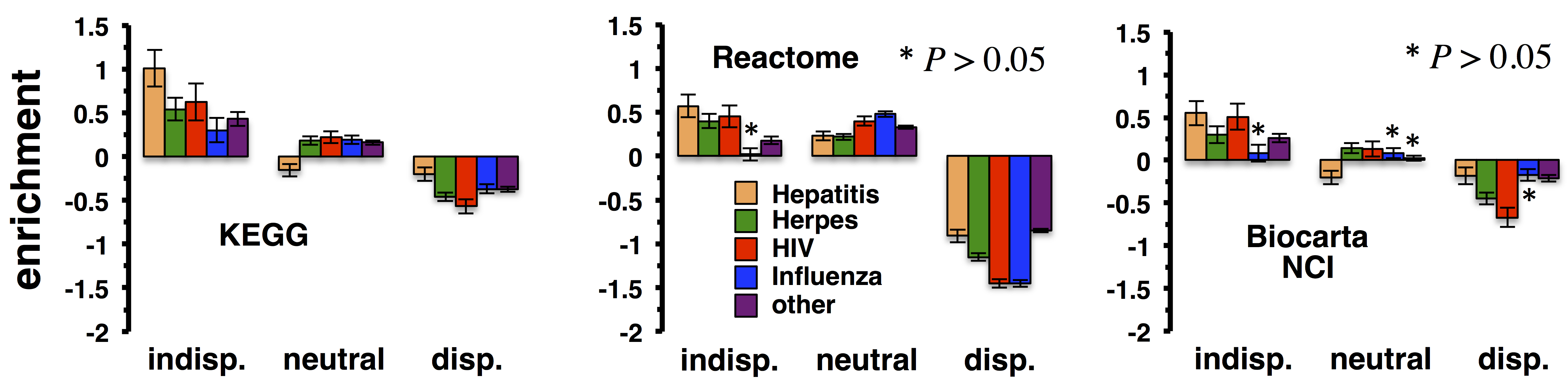

Supplement: S5 Fig — Randomizing sets of proteins that are targeted by Hepatitis, Herpes, HIV, Influenza and other viruses 10,000 times, we observed that indispensable proteins were preferably targeted by viruses (P<10−4) while the opposite held for dispensable nodes. Such observations held irrespective of the underlying pathway data source. (TIFF) [file pone.0197595.s005.tiff]

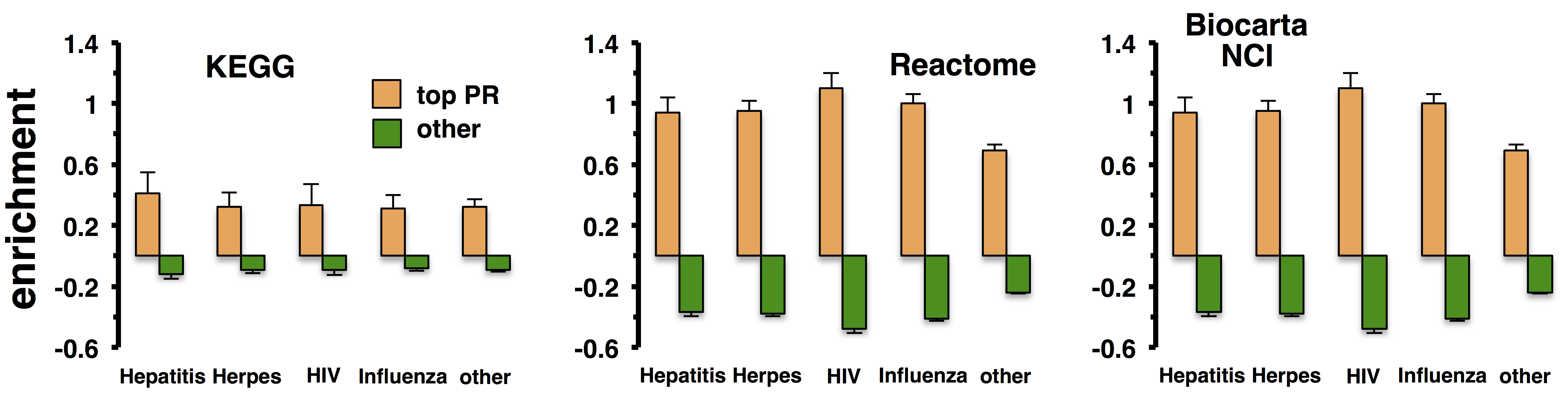

Supplement: S6 Fig — Randomizing sets of viral targets 10,000 times we determined their enrichment in sets of the top 20% of proteins with highest PageRank index. We observed that proteins with top PageRank were significantly enriched with viral targets and vice versa (P < 0.01). Notably, such observations were independent from pathway specific data. (TIFF) [file pone.0197595.s006.tiff]

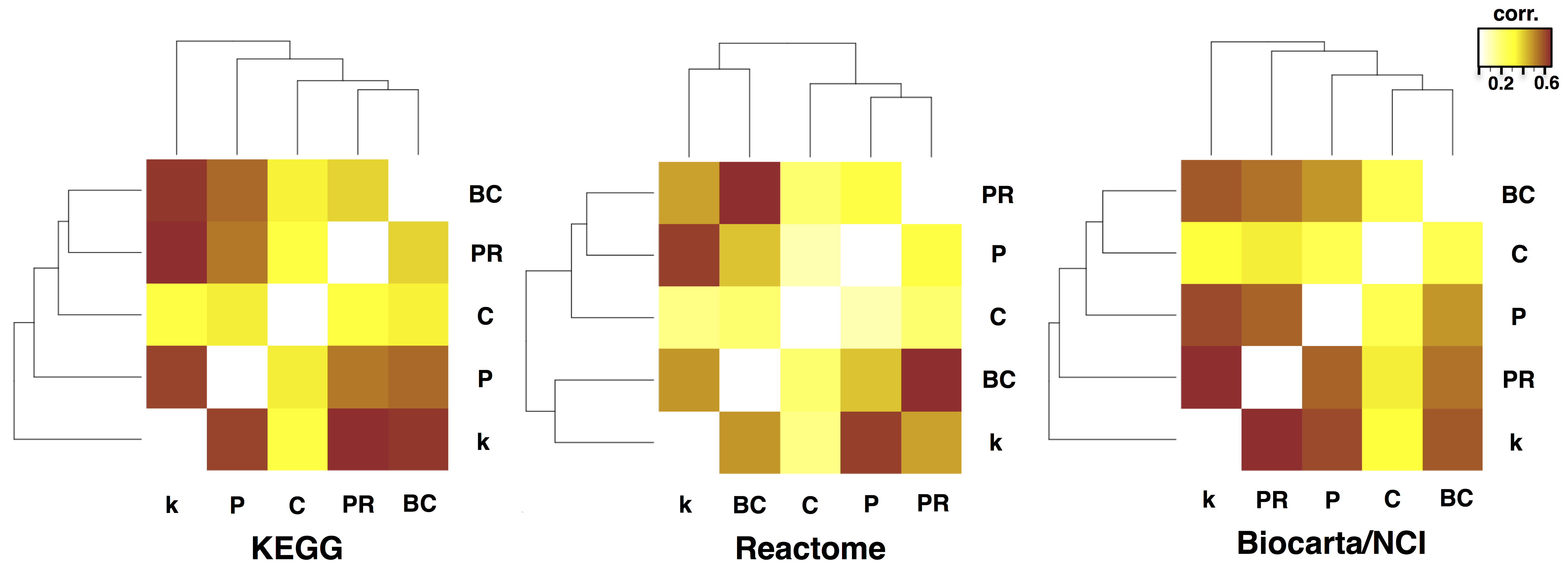

Supplement: S7 Fig — The heatmap indicated Pearson correlation values between the distributions of degree, betweenness centrality, number of pathways a protein is involved in, protein PageRank and indispensability of a protein using different pathway information. (TIFF) [file pone.0197595.s007.tiff]

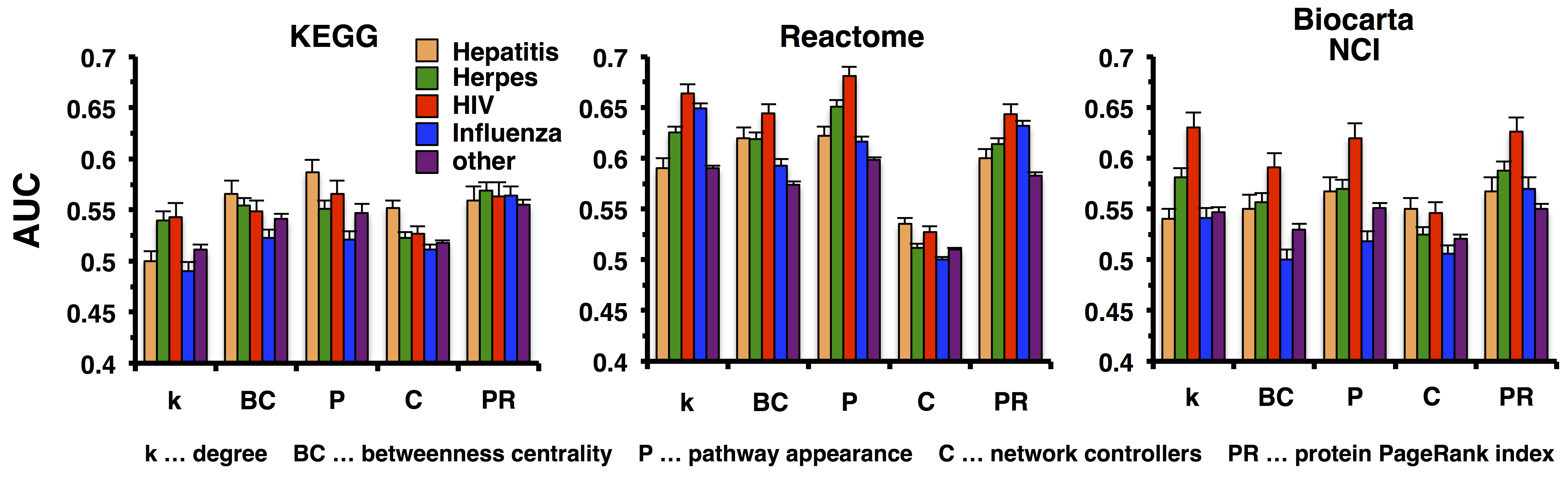

Supplement: S8 Fig — Considering target sets of Hepatitis, Herpes, HIV, Influenza and other viruses, we randomly sampled sets of non-targeted proteins of equal size. In comparison to betweenness centrality (BC) and protein’s indispensability (C) we observed that protein PageRank index (PR), pathway participation (P) of a protein and a proteins degree (k) allowed the most thorough classification of (non-)targets. Such observations were independent from the pathway information used. (TIFF) [file pone.0197595.s008.tiff]

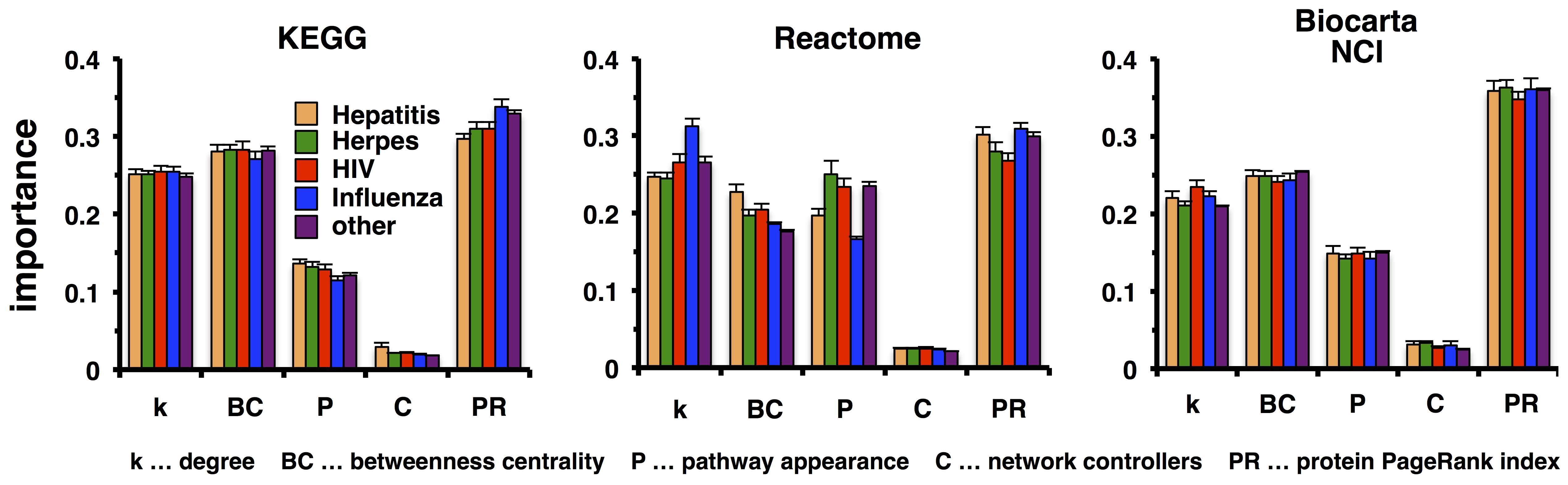

Supplement: S9 Fig — We utilized all five topological measures to predict viral targets using a random forest. We found that protein PageRank had the highest impact on the classification process, a result that was independent of the underlying virus and pathway information. (TIFF) [file pone.0197595.s009.tiff]

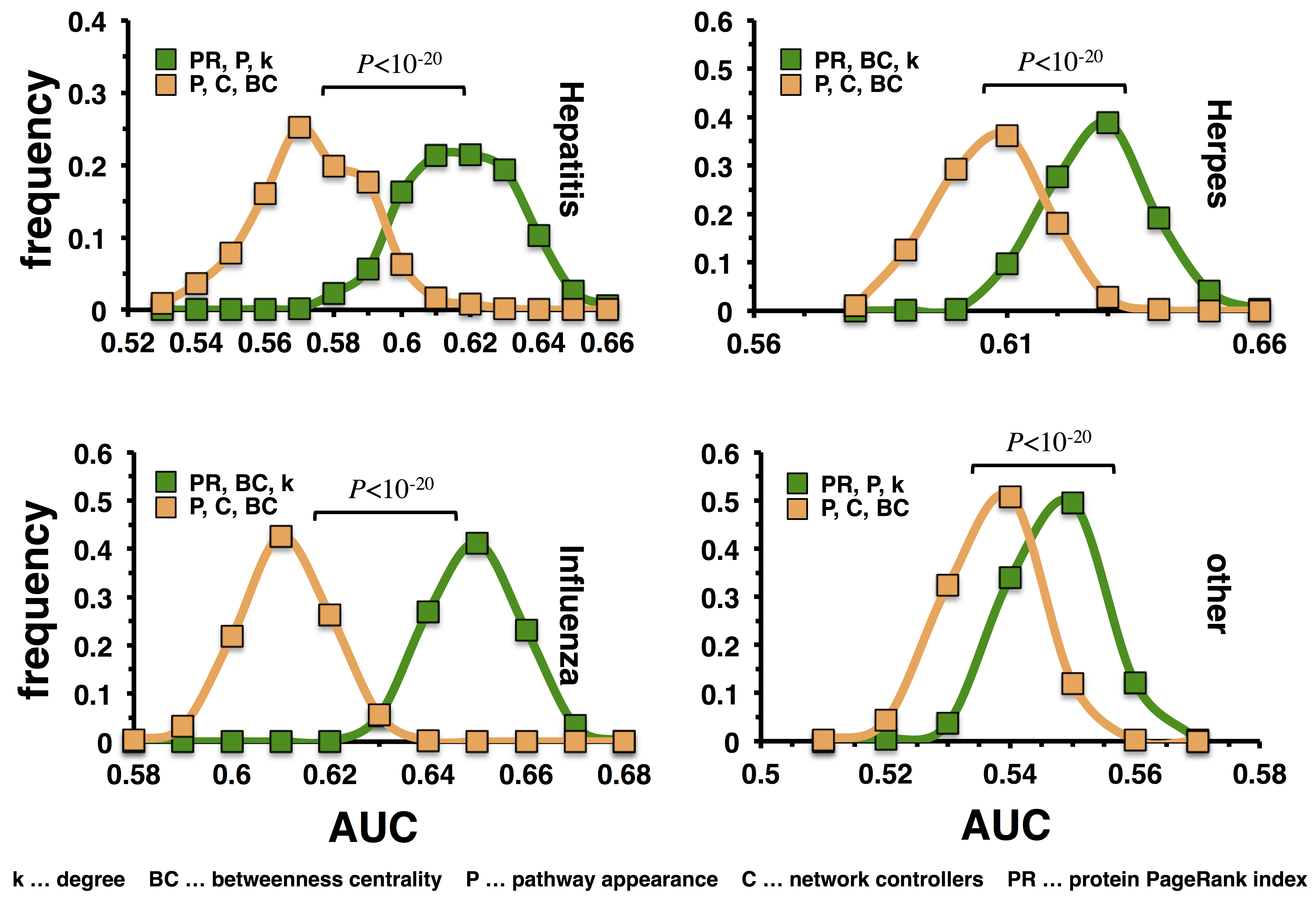

Supplement: S10 Fig — Focusing on proteins that are targeted by Hepatitis, Herpes, Influenza and other viruses, we randomly sampled non-targeted proteins of equal size 1,000 times. Furthermore, we used a random forest trained with the three most and least important features. In all cases, we found that the AUC curves obtained from the most important features allowed a significantly better classification (Student’s t-test, P < 10−20). (TIFF) [file pone.0197595.s010.tiff]
